# Treatment of schistosomiasis in African infants and preschool-aged children: downward extension and biometric optimization of the current praziquantel dose pole

**DOI:** 10.1016/j.inhe.2012.03.003

**Published:** 2012-06

**Authors:** José C. Sousa-Figueiredo, Martha Betson, J. Russell Stothard

**Affiliations:** a Disease Control Strategy Group, Liverpool School of Tropical Medicine, Pembroke Place, Liverpool, L3 5QA, United Kingdom; b Department of Infectious and Tropical Diseases, London School of Hygiene and Tropical Medicine, Keppel Street, London, WC1E 7HT, United Kingdom

**Keywords:** Child health, Neglected tropical diseases, Preventive chemotherapy, Mass drug administration, Tablet pole, Treatment pole

## Abstract

To facilitate administration of praziquantel (PZQ) to African infants and preschool-aged children using a dose pole, the performance of two downwardly extended versions (the first created in 2010 using biometric data from Uganda alone and the second version created here using data from 36 countries) was assessed against height/weight data from a total of 166 210 preschool-aged children (≤6 year olds) from 36 African countries. New and optimized thresholds for PZQ tablet administration at one tablet (600 mg), ¾ and ½ tablet divisions are suggested here. Both dose poles investigated estimated an acceptable PZQ dosage (30–60 mg/Kg) for more than 95% of children. Extension and optimization of the current PZQ dose pole, followed by theoretical validation using biometric data from preschool-aged children (0–6 years of age, 60–110 cm in height) from 36 African countries will help future mass drug administration campaigns incorporate younger children. This newly optimized dose pole with single 600 mg (height: 99–110 cm), ¾ (height: 83–99 cm) and ½ (height: 66–83 cm) tablet divisions, also reduces drug waste and facilitates inclusion of preschool-aged children. Our findings also have bearings on the use of other dose poles for treatment of young children.

## 1 Introduction

Since 2003, several mass drug administration (MDA) campaigns have been implemented in sub-Saharan Africa treating millions of school-aged children for schistosomiasis with praziquantel (PZQ). While these campaigns are having an enormous impact in reducing the morbidity suffered by millions of school-aged children, as an unforeseen consequence of current treatment guidelines and operational dosing tools, younger children (≤6 years) have been consistently excluded from access to such medication.^1^, ^2^, ^3^, ^4^

While the World Health Organization (WHO) and pharmaceutical sector consider treatment with PZQ as being safe for children as young as four years of age, this age-limit is not yet fully endorsed for ‘off-label’ use of PZQ in national control programmes settings.^5^, ^6^, ^7^, ^8^, ^9^ Nonetheless, as evidence-based advocacy grows this situation is set to change, for recent reports highlight that egg-patent infections can be found in children within the first year of life in high transmission settings.^3^, ^10^, ^11^, ^12^, ^13^, ^14^, ^15^, ^16^, ^17^, ^18^, ^19^, ^20^ Given the young child's risk of disease, this current health inequity should not persist. To this end, a significant programmatic realignment could be possible through development of a downward extension of the current WHO dosing pole from its present lowest height limit (i.e., 94 cm).^21^, ^22^, ^23^

The idea of dosing PZQ according to height instead of bodyweight (40 mg/Kg) was first investigated by Hall et al.^24^ Two years later, Montresor et al. expanded on this concept of using a simple and rudimentary height pole for pragmatic dosing of PZQ during school-based control campaigns.^25^ Since then the WHO dose pole, now revised to include children taller than 94 cm, has become essential in all campaigns delivering PZQ.^26^ To cater for the situation in Uganda where preschool-aged children are consistently shown to be at risk of infection and in need of treatment, the PZQ dose pole was extended downwards to include preschool-aged children (>60 cm tall) with height thresholds for administration of ¾ (84–99 cm) and ½ (60–84 cm) tablet divisions; a revised threshold for the administration of a single tablet (99–110 cm) was also developed owing to a previously poorer biometric model fit.^27^ Whilst this pole is now regularly used in Uganda, it awaits endorsement by WHO; theoretical testing using biometric information from young children in Angola (n = 1067), Mali (n = 405), Uganda (n = 3238), Sudan (n = 137), Zanzibar (n = 470) and Zimbabwe (n = 104) has led to good results, with more than 96% of children receiving acceptable dosages (30–60 mg/Kg).^28^, ^29^, ^30^, ^31^, ^32^ Nevertheless, more rigorous scrutiny of these new height divisions is needed to promote broader applicability and use in areas outside of Uganda.

The present study was conducted to investigate the theoretical validity of this extended PZQ dose pole in a far larger dataset representing 36 African countries. Importantly, we also used these data to establish a new biometric model, this time encompassing all 36 African countries (pan-African model) in an attempt to better capture inter-country variability in child growth (≤6 years of age, 55.0–129.9 cm tall, n *=* 175 276 children), against which we compared the initial Ugandan biometric model. The validation of both biometric models was conducted on height and weight data from 166 210 preschool-aged children (≤6 years of age, 60–110.0 cm tall).

## 2 Methods

In order to assemble a large cross-country data set various health agencies were first contacted with a request for collation of biometric data from infants and preschool-aged children across Africa, and with a brief description of how these data would be used.

### 2.1 Background information on praziquantel

Praziquantel is commonly marketed in 600 mg tablets and treatment is recommended using a regimen of 40 mg/Kg bodyweight in a single dose; an increased dosage of 60 mg/Kg can be advised in specific cases.^33^, ^34^ With PZQ tablets (600 mg) being available for as little as US$0.08 each, raw treatment costs per child have reduced dramatically.^33^, ^35^

Schistosome worms usually become susceptible to PZQ 6–8 weeks after infection and maturation within the host's body. Upon oral administration of PZQ, the drug acts quickly, typically within one hour, paralysing the worms and damaging their tegument facilitating immediate death or immune-dependent killing mechanisms. While there are reports of side-effects, these are generally mild and largely transient, although in exceptional cases progression towards general anaphylaxis can occur.^36^, ^37^ In certain situations, where disease transmission is high, a treatment protocol of two courses of PZQ (40 mg/Kg or higher) separated by 2–4 weeks is advocated but not yet fully endorsed by WHO as an alternative dosing approach to raise parasitological cure-rates.^38^, ^39^, ^40^ In terms of treatment of the younger child, as detailed pharmacokinetic studies are lacking yet the need for treatment is clear, a sensible approach would be to optimise dosing of young children at 40 mg/Kg, with an acceptable dose ranging from 30–60 mg/Kg until evidence to the contrary is presented.

### 2.2 Height and weight data

**Figure 1:**
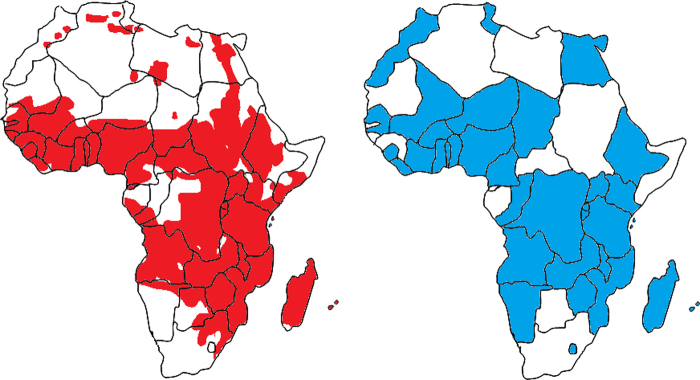
Maps illustrating schistosomiasis-endemic regions (left) and countries from which data were obtained (right). Information on the patterns of schistosomiasis endemicity was gathered from the maps presented by Schistosomiasis Research Group at Cambridge, UK (see http://www.path.cam.ac.uk/∼schisto/index.html).

Taking advantage of online databases compiled by MEASURE (a demographic health surveys data base http://www.measuredhs.com/start.cfm) population-based data were obtained on the age, gender, height and weight of children from 0–6 years of age from countries where schistosomiasis is endemic (see Figure 1). Data sets are described in Table 1. Height and weight were recorded to 0.1 cm and 0.1 Kg accuracy. For Angola and Morocco, weight was recorded to 1 Kg and 0.5 Kg accuracy, respectively. Height measurements in infants (<1 year olds) were conducted as the child was lying down (i.e., length). Z-scores were constructed using the WHO (Geneva, Switzerland) database for child growth standards (2006)*.* Quality of data was validated using Z-score calculation; implausible data were identified, double-checked and, if no mistakes were identified, implausible data were deleted from the database as they were probably the result of human error in the field or during data entry, i.e., weight-for-age Z-scores below –6 and above +5; height-for-age Z-scores below –6 above +6; weight-for-height and BMI-for-age Z-scores below –5 and above +5. Furthermore, only children aged between 6 and 72 months old (i.e., 55–129.9 cm tall) were included in the primary analysis in an attempt to establish a new pan-African model (data from all 36 countries). After all the criteria were met, the resulting sample size used to establish the pan-African model was 175 276 children (total censored 5914).

**Table 1:** Description of data sets analysed for standardised height-based praziquantel treatment schedule

Data set	Source population	Sample size (55-129.9 cm)	Sample size (60-110 cm)
Angola malaria, neglected tropical disease and malnutrition survey, 2010^a^	Angola Ages 1–72 months	1 067	992
Benin demographic and health survey-V, 2006^b^	Benin Ages 0–48 months	13 323	12 156
Burkina Faso demographic and health survey-IV, 2003^b^	Burkina Faso Ages 0–48 months	8 761	8 150
Burundi demographic and health survey-I, 1997^b^	Burundi Ages 0–36 months	1 936	1 857
Cameroon demographic and health survey-IV, 2004^b^	Cameroon Ages 0–48 months	3 317	3 061
Chad demographic and health survey-IV, 2004^b^	Chad Ages 0–48 months	4 636	4 324
Comoros Islands demographic and health survey-III, 1996^b^	Comoros Islands Ages 0–24 months	994	881
Congo (Brazzaville) demographic and health survey-V, 2005^b^	Congo (Brazzaville) Ages 0–48 months	4 054	3 709
Congo Democratic Republic demographic and health survey-V, 2007^b^	Congo DR Ages 0–48 months	3 564	3 287
Côte d’Ivoire demographic and health survey-III, 1998–1999^b^	Côte d’Ivoire Ages 0–48 months	1 586	1 480
Egypt demographic and health survey-V, 2005^b^	Egypt Ages 0–48 months	13 113	11 851
Ethiopia demographic and health survey-IV, 2000^b^	Ethiopia Ages 0–48 months	4 129	3 872
Ghana demographic and health survey-V, 2008^b^	Ghana Ages 0–48 months	2 482	2 345
Kenya demographic and health survey-V, 2008-2009^b^	Kenya Ages 0–48 months	5 315	4 927
Lesotho demographic and health survey-VI, 2009^b^	Lesotho Ages 0–48 months	1 674	1 540
Liberia demographic and health survey-V, 2007^b^	Liberia Ages 0–48 months	4 508	4 209
Madagascar demographic and health survey-IV, 2003–2004^b^	Madagascar Ages 0–48 months	4 691	4 287
Malawi demographic and health survey-IV, 2004^b^	Malawi Ages 0–48 months	8 573	7 877
Mali demographic and health survey-V, 2006^b^	Mali Ages 0–48 months	11 517	10 595
Morocco demographic and health survey-IV, 2003–2004^b^	Morocco Ages 0–48 months	5 643	5 260
Mozambique demographic and health survey-IV, 2003^b^	Mozambique Ages 0–48 months	8 252	7 487
Namibia demographic and health survey-V, 2006–2007^b^	Namibia Ages 0–48 months	3 802	3 496
Niger demographic and health survey-V, 2006^b^	Niger Ages 0–48 months	3 849	3 563
Nigeria demographic and health survey-V, 2008^b^	Nigeria Ages 0–48 months	21 850	19 701
Rwanda demographic and health survey-V, 2005^b^	Rwanda Ages 0–48 months	3 747	3 448
São Tomé & Príncipe demographic and health survey-V, 2008–2009^b^	São Tomé & Príncipe Ages 0–48 months	1 614	1 474
Senegal demographic and health survey-IV, 2005^b^	Senegal Ages 0–48 months	2 920	2 707
Sierra Leone demographic and health survey-V, 2008^b^	Sierra Leone Ages 0–48 months	2 250	2 058
Swaziland demographic and health survey-V, 2006–2007^b^	Swaziland Ages 0–48 months	2 085	1 955
Tanzania demographic and health survey-VI, 2010^b^	Tanzania Ages 0–48 months	6 919	6 396
Togo demographic and health survey-III, 1998^b^	Togo Ages 0–48 months	3 747	3 372
Tunisia demographic and health survey-I, 1988^b^	Tunisia Ages 0–36 months	2 033	1 978
Uganda schistosomiasis in mothers and infants (SIMI) project, 2009–2010^c^	Uganda Ages 6–72 months	3 302	2 683
Zambia demographic and health survey-V, 2007^b^	Zambia Ages 0–48 months	5 344	4 962
Zanzibar urinary schistosomiasis control programme monitoring, 2006^d^	Zanzibar Ages 0–72 months	470	443
Zimbabwe demographic and health survey-V, 2005-2006^b^	Zimbabwe Ages 0–48 months	4 121	3 827
TOTAL	Ages 0–72 months	175 276	166 210

a Centro de Investigação em Saúde em Angola (CISA), Bengo, Angola.

b Measure/DHS+, ORC Macro International, USA.

c Ugandan Ministry of Health and Natural History Museum (London).

d Zanzibar Ministry of Health and Natural History Museum (London).

### 2.3 Analysis

Zanzibari, Angolan and Ugandan biometric data were collected by the authors during investigative epidemiological surveys using pro-forma data sheets, which were then entered using EpiData (The EpiData Association, Odense, Denmark) or Microsoft Excel spreadsheet software (Microsoft Corp., Redmond, WA, USA).

For data compiled using MEASURE, biometric records were downloaded from the website and compiled using Stata version 11.0 (StataCorp, College Station, TX, USA), whereby the relevant columns were extracted into a Microsoft Excel file (.csv format): country code, gender, age, weight (in Kg), height (in mm), year of measurement and method of measurement (lying down or standing). The data thus collated were analysed using the R statistical package^®^ v 2·10·1 (The R Foundation for Statistical Computing, Vienna, Austria) and Microsoft Excel software. For percentage values (prevalence of children receiving a dose), 95% CIs were estimated using the exact method.^41^ Percentage of children receiving a certain dose according to both dose poles was compared using (one-tailed) Fisher's exact modification of the 2 × 2 χ^2^ test.^42^ Univariate models (height vs weight) were established (linear, polynomial, logarithmic and exponential) and model comparisons were conducted using Akaike information criterion (AIC).^43^ For more details on how models were established and compared see Hall et al.^24^

Once established, the original Ugandan (*weight* = 0.0029*x*^2^ − 0.2817*height* + 14.526) and the pan-African models (*weight* = 0.2268*height* – 7.6172) were theoretically applied to a subset of the initial population of preschool-aged children (0–6 years of age), this time ranging only from 60–110 cm tall.^27^ A 60 cm lower limit was used since this is the lower limit set by the current Ugandan model for ½ tablet administration.^27^, ^30^ An upper limit of 110 cm was used since this is the threshold established for 1½ tablets by the current WHO dose pole.^25^ All 36 datasets were unified into a single pan-African dataset and the resulting sample size was 166 210 children, i.e., 175 276 children (55.0–129.9 cm tall) made up the complete dataset used to establish the pan-African model and 166 210 children (60–110 cm tall) made up the subset on which both models were tested.

For each model, the following parameters were calculated: the mean dosage given (and standard deviation), the number and percentage of children receiving less than 30 mg/Kg (sub-curative dose), the number and percentage of children receiving 30–39 mg/Kg (sub-optimal but acceptable dosage), the number and percentage of children receiving 40–49 mg/Kg (optimal dose), the number and percentage of children receiving 50–60 mg/Kg (optimal dose), the number and percentage of children receiving more than 60 mg/Kg (overdosed).^31^, ^32^

## 3 Results

The Ugandan model was established using a dataset of 1046 Ugandan preschool-aged children.^27^ Using this model to estimate dose of PZQ according to the height of preschool-aged African children (n *=* 166 210) resulted in 58.2% (95% CI 58.0–58.5%) children receiving an optimum dosage (40–60 mg/Kg), while 95.3% (95% CI 95.2–95.4%) received an acceptable dosage (30–60 mg/Kg). Importantly, only 1.6% (95% CI 1.55–1.66%) of children would have received a sub-curative dosage (<30 mg/Kg), and 3.0% (95% CI 3.01–3.18%) would have received a slight over-dosage (>60 mg/Kg) (see Table 2).

**Table 2:** Performance of the models in estimating praziquantel dosages in 166 210 preschool-aged children (≤6 year olds) from 36 African countries (height range 60–110 cm). A praziquantel optimal dose was defined as being from 40–60 mg/Kg and an acceptable dosage as being from 30–60 mg/Kg

	Ugandan model	Pan-African model
Dose		
Average (SD) in mg/Kg	42.8 (7.8)	41.0 (6.8)

n (%) of people receiving:		
<30 mg/Kg^a^	2 672 (1.6)	4 494 (2.7)
30–39 mg/Kg^a^	61 614 (37.1)	74 316 (44.7)
40–49 mg/Kg^a^	80 267 (48.5)	76 084 (45.8)
50–60 mg/Kg^a^	16 506 (9.9)	8 529 (5.1)
>60 mg/Kg^a^	5 151 (3.0)	2 787 (1.7)
Acceptable dosage^a^	95.3%	95.6%
Optimal dose^a^	58.2%	50.9%

SD: standard deviation.

^a^ indicates the two performances were significantly different (p < 0.05).

The pan-African model was established using the complete dataset (55–129.9 cm, ages 6–72 months, n *=* 175 276 children). A linear model fitted to the height and weight data from all these children was found to be the most effective at describing the variability in the data: *weight* = 0.2268 *height* – 7.6172. Other univariate models attempted, which remained unselected by the AIC method, included polynomial, logarithmic and exponential (data not shown).

Using this model to estimate dosage of PZQ according to the height of the child (n *=* 166 210, height range 60.0–110.0 cm) resulted in 50.9% (95% CI 50.7–51.1%) children receiving an optimum dosage (40–60 mg/Kg), while 95.6% (95% CI 95.5–95.7%) received an acceptable dosage (30–60 mg/Kg). Of note is the fact that while the Ugandan model performed significantly better at estimating optimum dosages when compared to the pan-African model (58.2% vs 50.9%, respectively, p *<* 0.0001), the pan-African model was significantly better when considering an overall performance (95.6 vs 95.3%, p *<* 0.0001). Importantly, according to the pan-African model, only 2.7% (95% CI 2.62–2.78%) of children would have received a sub-curative dosage (<30 mg/Kg), and 1.7% (95% CI 1.62–1.74%) would have received a slight over-dosage (>60 mg/Kg) (see Table 2). These values are significantly different from those achieved by the Ugandan model, whereby the Ugandan model estimated significantly more over-dosage (3.0 vs 1.7%, p *<* 0.0001) and significantly fewer sub-curative dosages (1.6 vs 2.7%, p *<* 0.0001).

**Figure 2:**
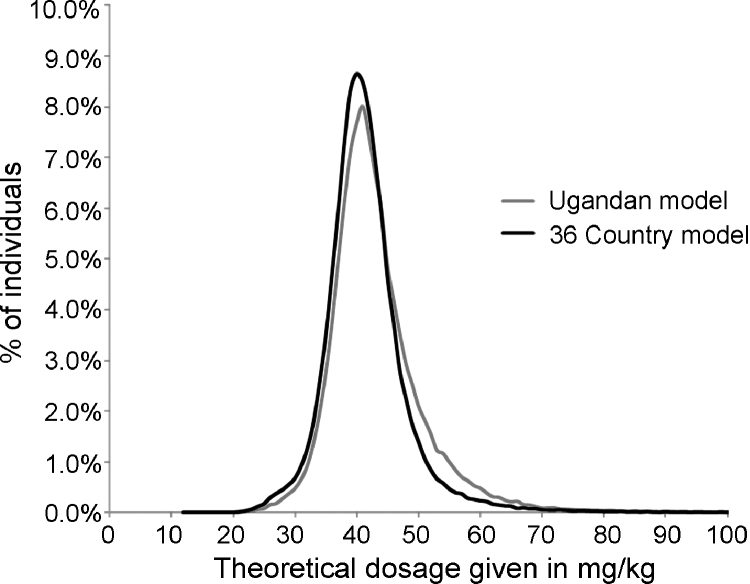
Distribution (percentage of children) of height-determined dosages for children aged 6–72 months receiving praziquantel tablets using a standardised schedule according to the two models tested (n *=* 166 210).

Figure 2 shows the distribution (percentage of children) of height-determined dosages achieved by both models (n *=* 166 210). Both data resemble normal distributions around the target of 40 mg/Kg, albeit skewed to the right for the Ugandan model indicating a minor tendency to slightly over-dose. Supplementary Table 1, Table 2 provide information on country- and region-specific performance of the Ugandan and pan-African models, respectively.

## 4 Discussion

As MDA-based control programmes are rolled-out throughout Africa offering PZQ, most of the monitoring and evaluation efforts, as well as applied scientific research, is being directed towards understanding pre- and post-treatment disease dynamics. If MDA is to become the principal way by which we combat schistosomiasis, and preschool-aged children are still excluded from the target demographics of current MDA efforts, these research and control activities are perpetuating a health inequality and failing to protect those most vulnerable to future ill-health and morbidity. New focus is therefore needed on understanding how schistosomiasis is already affecting younger children (<94 cm), developing appropriate control tools and promotion of formal targeting within current control programmes, especially where programmatic integration with control of other neglected tropical diseases is needed.^3^, ^10^, ^11^, ^12^, ^13^, ^14^, ^15^, ^16^, ^17^, ^18^, ^19^, ^20^ An applied research agenda which perhaps should have benefited by the attention of SCORE (Schistosomiasis Consortium for Operational Research and Evaluation - see http://score.uga.edu/) that aimed to answer strategic questions about schistosomiasis control and elimination, but an opportunity unfortunately now missed.

### 4.1 An extended praziquantel dose pole

**Figure 3:**
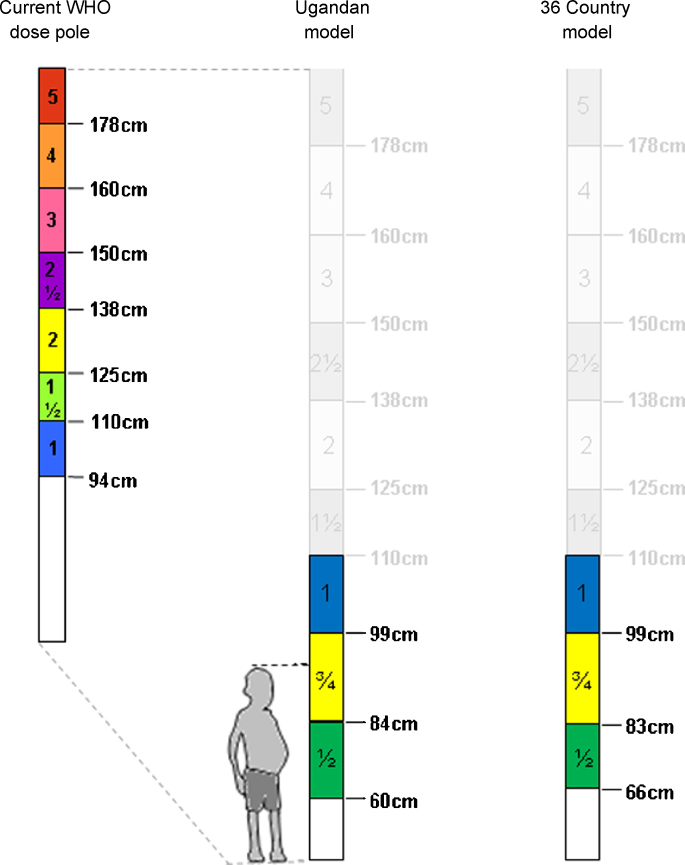
Pictorial representation of the current WHO dose pole for administration of praziquantel tablets (at 600 mg each) (left) and the new dose poles - Ugandan model (centre) and pan-African model (right) with new height thresholds added to allow for treatment of preschool-aged children (<6 year olds). Additionally, the WHO pole's single tablet lower limit has been corrected from 94 cm to 99 cm by both models. The illustrated child needs administration of a ¾ tablet division rather than a single tablet.

This study suggests that in order to permit inclusion of younger children in pragmatic dosing of PZQ, downward extension of the current dose pole is required. The two biometric models tested here have their strengths and weaknesses. While the Ugandan model is capable of predicting more optimum dosages (58.2% of children receiving 40–60 mg/Kg), the pan-African model correctly predicted more acceptable dosing (95.6% of children receiving 30–60 mg/Kg) covering a range from 6-months to 6 years of age. Importantly, even though the genesis populations differ in size (n *=* 1046 for the first and n *=* 175 276 for the second) and origin, both models result in fairly similar dose poles. In fact, both models support the need for an upward revision of the current single tablet lower threshold from 94 cm to 99 cm.^27^, ^30^ According to the Ugandan model, the height-intervals for ½ tablet and ¾ tablet divisions should be 60–84 cm and 84–99 cm, corresponding to a mean age of 1 year and 2¾ years, respectively.^27^ The 36-country model, on the other hand, corrected the lower limit for ¾ tablet division (now 83–99 cm) and raised the lower height-limit eligible for treatment using a dose pole (66–83 cm for ½ tablet division). For a pictorial representation of the current WHO dose pole, as well as the dose poles predicted by the two models tested here see Figure 3.

Choosing which model, i.e., which dose pole, should be implemented requires a discussion of selection criteria. For example, one is capable of predicting more optimum dosages (Ugandan model), while the other performs better overall (pan-African model) across assembled countries. However, at the international level we propose that the pan-African model is the favoured option since its genesis population is far larger and more representative of the pan-African population, which will be treated by any future extended dose pole to be endorsed by WHO. The pan-African model presented here does not adjust for any variation in the height data between countries; this variation is implied in the database, and by considering all countries as one single database we hoped to control implicitly for this variation. Of note is the fact that both models have been tested for each country individually and both models were found to perform significantly differently for each region of Africa (Supplementary Tables 1 and 2). More particularly, each model performed similarly in Southern, Central and Eastern Africa, while showing very different performances for Western and Northern Africa.

### 4.2 Health and economic impact of a revised dose pole

Without a doubt, treating children at a younger age with PZQ can lead to significant decreases in overall infection intensities, as well as prevalence levels in children as they progress to school-age.^29^ This is important considering the purpose of current control programmes (i.e., morbidity control: dampening the occurrence of raised infection intensities). Therefore treatment of children at an earlier age can result in earlier reductions in egg-counts and disease-progression.^29^, ^44^ Also, by negating natural transmission from an earlier age and preventing high worm burdens, treatment of younger children could lead to long-term economic savings as younger children require fewer tablets. Treating younger children could be a sensible future strategic realignment of MDA, building for longer-term sustainability over forthcoming decades, as the treatment needs in older children are set to decline.

There are also immediate economic savings to be considered if the current WHO dose pole is revised to start treatment with a single tablet at 99 cm and not the current 94 cm. Pharmaceutical companies are now engaged to provide large tablet procurements/donations to control programmes (see London Declaration on Neglected Tropical Diseases in http://www.unitingtocombatntds.org/), meaning drug wastage has become an important issue, and using the new height threshold of 99 cm could lead to significant savings of tablets (and money).^29^, ^30^ In our sample of 166 210 children, 11.3% (18 806) were between 94 and 99 cm tall (ages between 2 and 5 years, mean age of 3.9 years). Using the corrected height threshold (99 cm) rather than the original one (94 cm) would have resulted in saving some 4700 tablets. While this level of drug wastage might appear negligible, extrapolating this continent-wide reveals its importance, with millions of children currently living in endemic areas.

## 5 Conclusions

Following on from a comprehensive biometric investigation, two models for creation of an extended PZQ dose pole for younger children were validated against an extensive database of 166 210 preschool-aged children (≤6 years of age, 60.0–110.0 cm tall) from 36 countries in Africa. Downward extension and optimization of present height thresholds of the current WHO dose pole for administration of a single 600 mg tablet (height: 99–110 cm), as well as ¾ (height: 83–99 cm) and ½ (height: 66–83 cm) tablet divisions reduces drug waste and allows for inclusion of preschool-aged children during MDA campaigns.

## Authors’ disclaimers

The authors alone are responsible for the views expressed in this article, which might not necessarily reflect the opinion or policy of their employing institutions.

## Authors’ contributions

JRS and JCSF conceived and designed the study; JCSF analysed the data; JCSF, MB and JRS interpreted the data; JCSF and JRS drafted the manuscript; JCSF, MB and JRS revised the manuscript for intellectual content, and read and approved the final version. JRS is guarantor of the paper.

## Funding

This work was supported by a project grant awarded to JRS by the Wellcome Trust, UK: Gibbs Building, 215 Euston Road, London NW1 2BE, UK.

## Competing interests

None declared.

## Ethical approval

The Ugandan National Council of Science and Technology and the London School of Hygiene and Tropical Medicine, UK, granted ethical approval for the studies conducted in Uganda (application nos. LSHTM 06·45 and LSHTM 5538·09).
