# Supplementary material for: Treatment of schistosomiasis in African infants and preschool-aged children: downward extension and biometric optimization of the current praziquantel dose pole
Source: Int Health. 2012 Jun;4(2):95–102. doi: 10.1016/j.inhe.2012.03.003 (PMC3407873; doi:10.1016/j.inhe.2012.03.003)
Supplement: Supplementary file 1 [file mmc1.doc]

**Supplementary Table 1**. Performance of the Ugandan model (*weight*= 0·0029*x*^2^ – 0·2817 · *height* + 14·526) in estimating praziquantel dosages in 166 210 preschool-aged children (≤6 year olds; height range 60–110cm) from 36 African countries

|  |  | **Dose**  **administered** | **No. (%) of individuals receiving dose** | | | | | | | | | |
| --- | --- | --- | --- | --- | --- | --- | --- | --- | --- | --- | --- | --- |
| **Country** | **n** | **Average (SD)**  **in mg/kg** | **<30 mg/kg** | | **≥30 and <40**  **mg/kg** | | **≥40 and <50**  **mg/kg** | | **≥50 to <60**  **mg/kg** | | **>60 mg/kg** | |
|  |  |  | **n** | **%** | **n** | **%** | **n** | **%** | **n** | **%** | **n** | **%** |
| Angola | 992 | 41.9 (5.0) | 6 | 0.6% | 359 | 36.2% | 572 | 57.7% | 50 | 5.0% | 5 | 0.5% |
| Benin | 12 156 | 41.9 (7.2) | 293 | 2.4% | 4752 | 39.1% | 5785 | 47.6% | 1091 | 9.0% | 235 | 1.9% |
| Burkina Faso | 8150 | 46.1 (10.5) | 104 | 1.3% | 2021 | 24.8% | 3992 | 49.0% | 1378 | 16.9% | 655 | 8.0% |
| Burundi | 1857 | 44.6 (5.9) | 3 | 0.2% | 372 | 20.0% | 1184 | 63.8% | 265 | 14.3% | 33 | 1.8% |
| Cameroon | 3061 | 41.2 (6.7) | 32 | 1.0% | 1443 | 47.1% | 1333 | 43.5% | 214 | 7.0% | 39 | 1.3% |
| Chad | 4324 | 45.0 (8.4) | 46 | 1.1% | 1055 | 24.4% | 2327 | 53.8% | 682 | 15.8% | 214 | 4.9% |
| Comoros Isl. | 881 | 44.4 (8.2) | 4 | 0.5% | 268 | 30.4% | 433 | 49.1% | 140 | 15.9% | 36 | 4.1% |
| Congo, Republic of the | 3709 | 42.0 (7.0) | 43 | 1.2% | 1548 | 41.7% | 1740 | 46.9% | 298 | 8.0% | 80 | 2.2% |
| Côte d’Ivoire | 1480 | 42.3 (6.2) | 18 | 1.2% | 543 | 36.7% | 771 | 52.1% | 131 | 8.9% | 17 | 1.1% |
| DRC | 3287 | 42.8 (7.7) | 73 | 2.2% | 1124 | 34.2% | 1666 | 50.7% | 323 | 9.8% | 101 | 3.1% |
| Egypt | 11 851 | 39.6 (6.4) | 436 | 3.7% | 6497 | 54.8% | 4250 | 35.9% | 529 | 4.5% | 139 | 1.2% |
| Ethiopia | 3872 | 44.1 (7.8) | 45 | 1.2% | 1010 | 26.1% | 2240 | 57.9% | 441 | 11.4% | 136 | 3.5% |
| Ghana | 2345 | 42.9 (7.7) | 30 | 1.3% | 778 | 33.2% | 1265 | 53.9% | 218 | 9.3% | 54 | 2.3% |
| Kenya | 4927 | 42.5 (6.7) | 49 | 1.0% | 1772 | 36.0% | 2604 | 52.9% | 405 | 8.2% | 97 | 2.0% |
| Lesotho | 1540 | 41.5 (6.7) | 17 | 1.1% | 678 | 44.0% | 729 | 47.3% | 97 | 6.3% | 19 | 1.2% |
| Liberia | 4209 | 42.6 (7.0) | 41 | 1.0% | 1629 | 38.7% | 2057 | 48.9% | 394 | 9.4% | 88 | 2.1% |
| Madagascar | 4287 | 44.2 (7.0) | 39 | 0.9% | 986 | 23.0% | 2568 | 59.9% | 579 | 13.5% | 115 | 2.7% |
| Malawi | 7877 | 41.5 (7.9) | 122 | 1.5% | 3765 | 47.8% | 3214 | 40.8% | 601 | 7.6% | 175 | 2.2% |
| Mali | 10 595 | 44.7 (8.4) | 175 | 1.7% | 2608 | 24.6% | 5774 | 54.5% | 1543 | 14.6% | 495 | 4.7% |
| Morocco | 5260 | 41.3 (8.1) | 133 | 2.5% | 2592 | 49.3% | 1982 | 37.7% | 402 | 7.6% | 151 | 2.9% |
| Mozambique | 7487 | 41.7 (6.3) | 32 | 0.4% | 3332 | 44.5% | 3451 | 46.1% | 558 | 7.5% | 114 | 1.5% |
| Namibia | 3496 | 43.4 (6.8) | 23 | 0.7% | 966 | 27.6% | 2119 | 60.6% | 312 | 8.9% | 76 | 2.2% |
| Niger | 3563 | 44.4 (7.0) | 20 | 0.6% | 849 | 23.8% | 2133 | 59.9% | 445 | 12.5% | 116 | 3.3% |
| Nigeria | 19 701 | 44.0 (9.6) | 459 | 2.3% | 6907 | 35.1% | 8498 | 43.1% | 2507 | 12.7% | 1330 | 6.8% |
| Rwanda | 3448 | 41.2 (5.9) | 29 | 0.8% | 1645 | 47.7% | 1475 | 42.8% | 262 | 7.6% | 37 | 1.1% |
| Senegal | 1474 | 42.3 (7.9) | 46 | 1.7% | 532 | 19.7% | 722 | 26.7% | 121 | 4.5% | 53 | 2.0% |
| São Tomé e Príncipe | 2707 | 43.6 (5.9) | 8 | 0.4% | 675 | 32.8% | 1724 | 83.8% | 249 | 12.1% | 51 | 2.5% |
| Sierra Leone | 2058 | 42.6 (8.6) | 96 | 6.5% | 690 | 46.8% | 993 | 67.4% | 210 | 14.2% | 69 | 4.7% |
| Swaziland | 1955 | 39.9 (5.8) | 33 | 1.7% | 1125 | 57.5% | 700 | 35.8% | 83 | 4.2% | 14 | 0.7% |
| Tanzania | 6396 | 42.3 (6.1) | 30 | 0.5% | 2336 | 36.5% | 3483 | 54.5% | 458 | 7.2% | 89 | 1.4% |
| Togo | 3372 | 45.7 (7.2) | 11 | 0.3% | 667 | 19.8% | 1919 | 56.9% | 655 | 19.4% | 120 | 3.6% |
| Tunisia | 1978 | 41.9 (5.8) | 15 | 0.8% | 781 | 39.5% | 1015 | 51.3% | 143 | 7.2% | 24 | 1.2% |
| Uganda | 2683 | 40.7 (5.2) | 14 | 0.5% | 1335 | 49.7% | 1206 | 44.9% | 105 | 3.9% | 23 | 0.9% |
| Zambia | 4962 | 41.5 (6.5) | 69 | 1.4% | 2179 | 43.9% | 2298 | 46.3% | 338 | 6.8% | 78 | 1.6% |
| Zanzibar | 443 | 41.6 (5.5) | 5 | 1.1% | 156 | 35.3% | 257 | 58.1% | 21 | 4.8% | 4 | 0.9% |
| Zimbabwe | 3827 | 41.5 (6.6) | 73 | 1.9% | 1639 | 42.8% | 1788 | 46.7% | 258 | 6.7% | 69 | 1.8% |
| Northern Africa^a^ | 19 089 | 40.3 (6.9) | 584 | 3.1% | 9870 | 51.7% | 7247 | 38.0% | 1074 | 5.6% | 314 | 1.6% |
| Eastern Africa^b^ | 52 947 | 42.2 (6.8) | 514 | 1.0% | 20 795 | 39.3% | 26 201 | 49.5% | 4431 | 8.4% | 1006 | 1.9% |
| Western Africa^c^ | 70 336 | 43.9 (8.6) | 1255 | 1.8% | 22 119 | 31.4% | 34 911 | 49.6% | 8821 | 12.5% | 3230 | 4.6% |
| Southern and Central Africa^d^ | 23 838 | 42.6 (7.4) | 319 | 1.3% | 8830 | 37.0% | 11 908 | 50.0% | 2180 | 9.1% | 601 | 2.5% |
| **TOTAL (36 countries)** | **166210** | **42.7 (7.0)** | **2672** | **1.6%** | **61614** | **37.1%** | **80267** | **48.5%** | **16506** | **9.9%** | **5151** | **3.0%** |

Africa was divided according to the United Nation geoscheme (<http://en.wikipedia.org/wiki/United_Nations_geoscheme_for_Africa>):

^a^Northern Africa here included Egypt, Morocco and Tunisia;

^b^Eastern Africa here included Burundi, Comoros Islands, Ethiopia, Kenya, Madagascar, Malawi, Mozambique, Rwanda, Tanzania, Uganda, Zambia, Zanzibar and Zimbabwe;

^c^Western African here included Benin, Burkina Faso, Côte d’Ivoire, Ghana, Liberia, Mali, Niger, Nigeria, Senegal, Sierra Leone and Togo;

^d^Southern and Central Africa here included Angola, Cameroon, Chad, Republic of the Congo, Democratic Republic of the Congo (DRC), Lesotho, Namibia, São Tomé e Príncipe and Swaziland

Supplementary Table 2. Performance of the pan-African model (*weight* = 0.2268 · *height* – 7.6172) in estimating praziquantel dosages in 166 210 preschool-aged children (≤6 year olds; height range 60–110cm) from 36 African countries

|  |  | **Dose administered** | **No. (%) of individuals receiving dose** | | | | | | | | | |
| --- | --- | --- | --- | --- | --- | --- | --- | --- | --- | --- | --- | --- |
| **Country** | **n** | **Average (SD) in mg/kg** | **<30 mg/kg** | | **≥30 and <40 mg/kg** | | **≥40 and <50 mg/kg** | | **≥50 to <60 mg/kg** | | **>60 mg/kg** | |
|  |  |  | **n** | **%** | **n** | **%** | **n** | **%** | **n** | **%** | **n** | **%** |
| Angola | 992 | 41.1 (4.7) | 8 | 0.8% | 422 | 42.5% | 528 | 53.2% | 32 | 3.2% | 2 | 0.2% |
| Benin | 12 156 | 40.1 (6.4) | 587 | 4.8% | 5574 | 45.9% | 5383 | 44.3% | 502 | 4.1% | 110 | 0.9% |
| Burkina Faso | 8150 | 44.1 (8.9) | 159 | 2.0% | 2365 | 29.0% | 4261 | 52.3% | 1004 | 12.3% | 361 | 4.4% |
| Burundi | 1857 | 41.9 (4.4) | 7 | 0.4% | 587 | 31.6% | 1197 | 64.5% | 56 | 3.0% | 10 | 0.5% |
| Cameroon | 3061 | 39.5 (5.6) | 46 | 1.5% | 1761 | 57.5% | 1140 | 37.2% | 98 | 3.2% | 16 | 0.5% |
| Chad | 4324 | 42.9 (7.0) | 69 | 1.6% | 1343 | 31.1% | 2449 | 56.6% | 364 | 8.4% | 99 | 2.3% |
| Comoros Isl. | 881 | 41.6 (6.6) | 15 | 1.7% | 379 | 43.0% | 422 | 47.9% | 51 | 5.8% | 14 | 1.6% |
| Congo, Republic of the | 3709 | 40.3 (6.0) | 75 | 2.0% | 1839 | 49.6% | 1609 | 43.4% | 142 | 3.8% | 44 | 1.2% |
| Côte d’Ivoire | 1480 | 40.7 (5.3) | 27 | 1.8% | 656 | 44.3% | 749 | 50.6% | 42 | 2.8% | 6 | 0.4% |
| DRC | 3287 | 41.2 (6.9) | 127 | 3.9% | 1277 | 38.9% | 1642 | 50.0% | 186 | 5.7% | 55 | 1.7% |
| Egypt | 11 851 | 38.2 (5.7) | 710 | 6.0% | 7287 | 61.5% | 3533 | 29.8% | 257 | 2.2% | 64 | 0.5% |
| Ethiopia | 3872 | 42.2 (6.6) | 76 | 2.0% | 1308 | 33.8% | 2203 | 56.9% | 222 | 5.7% | 63 | 1.6% |
| Ghana | 2345 | 41.4 (6.6) | 47 | 2.0% | 901 | 38.4% | 1282 | 54.7% | 93 | 4.0% | 22 | 0.9% |
| Kenya | 4927 | 40.9 (5.9) | 81 | 1.6% | 2216 | 45.0% | 2409 | 48.9% | 168 | 3.4% | 53 | 1.1% |
| Lesotho | 1540 | 40.0 (6.0) | 33 | 2.1% | 819 | 53.2% | 636 | 41.3% | 43 | 2.8% | 9 | 0.6% |
| Liberia | 4209 | 40.9 (5.9) | 54 | 1.3% | 1936 | 46.0% | 2011 | 47.8% | 163 | 3.9% | 45 | 1.1% |
| Madagascar | 4287 | 42.4 (6.1) | 94 | 2.2% | 1221 | 28.5% | 2634 | 61.4% | 280 | 6.5% | 58 | 1.4% |
| Malawi | 7877 | 39.5 (6.6) | 222 | 2.8% | 4584 | 58.2% | 2702 | 34.3% | 265 | 3.4% | 104 | 1.3% |
| Mali | 10 595 | 42.9 (7.2) | 227 | 2.1% | 3244 | 30.6% | 5985 | 56.5% | 880 | 8.3% | 259 | 2.4% |
| Morocco | 5260 | 39.8 (7.4) | 224 | 4.3% | 2912 | 55.4% | 1727 | 32.8% | 305 | 5.8% | 92 | 1.7% |
| Mozambique | 7487 | 40.0 (5.0) | 58 | 0.8% | 4115 | 55.0% | 3084 | 41.2% | 180 | 2.4% | 50 | 0.7% |
| Namibia | 3496 | 41.5 (5.8) | 39 | 1.1% | 1275 | 36.5% | 2035 | 58.2% | 113 | 3.2% | 34 | 1.0% |
| Niger | 3563 | 42.4 (5.6) | 32 | 0.9% | 1108 | 31.1% | 2191 | 61.5% | 188 | 5.3% | 44 | 1.2% |
| Nigeria | 19 701 | 42.0 (8.6) | 794 | 4.0% | 8280 | 42.0% | 8078 | 41.0% | 1679 | 8.5% | 870 | 4.4% |
| Rwanda | 3448 | 39.5 (4.5) | 45 | 1.3% | 1977 | 57.3% | 1343 | 39.0% | 76 | 2.2% | 7 | 0.2% |
| Senegal | 1474 | 40.9 (7.5) | 71 | 4.8% | 631 | 42.8% | 638 | 43.3% | 96 | 6.5% | 38 | 2.6% |
| São Tomé e Príncipe | 2707 | 42.9 (4.7) | 14 | 0.5% | 917 | 33.9% | 1660 | 61.3% | 96 | 3.5% | 20 | 0.7% |
| Sierra Leone | 2058 | 40.9 (7.9) | 137 | 6.7% | 801 | 38.9% | 953 | 46.3% | 121 | 5.9% | 46 | 2.2% |
| Swaziland | 1955 | 38.4 (4.9) | 47 | 2.4% | 1344 | 68.7% | 527 | 27.0% | 25 | 1.3% | 12 | 0.6% |
| Tanzania | 6396 | 40.7 (5.2) | 55 | 0.9% | 2931 | 45.8% | 3191 | 49.9% | 176 | 2.8% | 43 | 0.7% |
| Togo | 3372 | 43.1 (5.5) | 25 | 0.7% | 920 | 27.3% | 2138 | 63.4% | 252 | 7.5% | 37 | 1.1% |
| Tunisia | 1978 | 40.1 (4.2) | 20 | 1.0% | 1014 | 51.3% | 912 | 46.1% | 28 | 1.4% | 4 | 0.2% |
| Uganda | 2683 | 39.7 (4.9) | 20 | 0.7% | 1555 | 58.0% | 1025 | 38.2% | 68 | 2.5% | 15 | 0.6% |
| Zambia | 4962 | 39.9 (5.6) | 121 | 2.4% | 2642 | 53.2% | 2007 | 40.4% | 154 | 3.1% | 38 | 0.8% |
| Zanzibar | 443 | 40.5 (5.4) | 5 | 1.1% | 193 | 43.6% | 227 | 51.2% | 16 | 3.6% | 2 | 0.5% |
| Zimbabwe | 3827 | 39.9 (5.8) | 123 | 3.2% | 1982 | 51.8% | 1573 | 41.1% | 108 | 2.8% | 41 | 1.1% |
| Northern Africa^a^ | 19 089 | 38.8 (6.1) | 954 | 5.0% | 11 213 | 58.7% | 6172 | 32.3% | 590 | 3.1% | 160 | 0.8% |
| Eastern Africa^b^ | 52 947 | 40.5 (5.8) | 922 | 1.7% | 25 690 | 48.5% | 24 017 | 45.4% | 1820 | 3.4% | 498 | 0.9% |
| Western Africa^c^ | 70 336 | 41.9 (7.5) | 2103 | 3.0% | 26 702 | 38.0% | 34 691 | 49.3% | 5020 | 7.1% | 1820 | 2.6% |
| Southern and Central Africa^d^ | 23 838 | 40.9 (6.4) | 515 | 2.2% | 10 711 | 44.9% | 11 204 | 47.0% | 1099 | 4.6% | 309 | 1.3% |
| **TOTAL (36 countries)** | **166210** | **41.0 (6.8)** | **4494** | **2.7%** | **74316** | **44.7%** | **76084** | **45.8%** | **8529** | **5.1%** | **2787** | **1.7%** |

Africa was divided according to the United Nation geoscheme (<http://en.wikipedia.org/wiki/United_Nations_geoscheme_for_Africa>):

^a^Northern Africa here included Egypt, Morocco and Tunisia

^b^Eastern Africa here included Burundi, Comoros Islands, Ethiopia, Kenya, Madagascar, Malawi, Mozambique, Rwanda, Tanzania, Uganda, Zambia, Zanzibar and Zimbabwe

^c^Western African here included Benin, Burkina Faso, Côte d’Ivoire, Ghana, Liberia, Mali, Niger, Nigeria, Senegal, Sierra Leone and Togo

^d^Southern and Central Africa here included Angola, Cameroon, Chad, Republic of the Congo, Democratic Republic of the Congo (DRC), Lesotho, Namibia, São Tomé e Príncipe and Swaziland
